# The Use of a Brief Antenatal Lifestyle Education Intervention to Reduce Preterm Birth: A Retrospective Cohort Study

**DOI:** 10.3390/nu14142799

**Published:** 2022-07-07

**Authors:** Na Wang, Jie Lu, Yan Zhao, Yuan Wei, Jenny Gamble, Debra K. Creedy

**Affiliations:** 1 School of Nursing, Capital Medical University, 10 Xitoutiao Road, Fengtai District, Beijing 100069, China; na_wang_3@163.com; 2 Department of Gynaecology and Obstetrics, Peking University Third Hospital, 49 Huayuan Beilu Road, Haidian District, Beijing 100191, China; zhaoyan_bysy@sina.com (Y.Z.); weiyuanbysy@163.com (Y.W.); 3 School of Nursing and Midwifery, Griffith University Logan Campus, University Drive, Meadowbrook, Logan, QLD 4131, Australia; j.gamble@griffith.edu.au (J.G.); d.creedy@griffith.edu.au (D.K.C.)

**Keywords:** antenatal education, lifestyle factors, preterm birth, intervention timing, health promotion

## Abstract

Preterm birth is a leading cause of neonatal and child mortality and morbidity worldwide. The aim of this study was to investigate associations between attending a brief antenatal lifestyle education seminar and preterm birth, and whether education timing modifies outcomes. A retrospective cohort study was conducted in a hospital-based antenatal care center in Beijing, China, where a free, 2 h, optional, face-to-face, midwife-led group seminar on healthy lifestyle choices during pregnancy was provided. Among the 3008 eligible women, 1107 (36.8%) attended the seminar during the first trimester, 515 (17.1%) attended during the second trimester or later, and 1386 (46.1%) did not attend. Multiparous women were more likely to not attend or to attend at a later stage. The overall prevalence of preterm birth was 8.7%, but it was higher for women who did not attend the antenatal seminar (11.5%). The risk of preterm birth for first trimester attendees decreased by 53%, and it decreased by 41% for later attendees. Estimates persisted after adjusting pre-existing and gestational covariates. Attending a brief antenatal lifestyle education seminar was associated with lower preterm birth risk, and attending during the first trimester had a better impact than later attendance. The results can inform the development of tailored preterm birth prevention strategies.

## 1. Introduction

The estimated worldwide incidence of preterm birth (defined as birth before 37 weeks of gestational age) is 11% [1]. Preterm birth is considered a leading cause of neonatal and child mortality and morbidity in low, middle and high-income countries [2,3,4]. Preterm birth prevention strategies have mainly focused on three areas: (1) optimizing neonatal care practices to improve preterm birth outcomes [5], (2) managing identified risk factors to reduce threatened or underlying preterm birth [6] and (3) promoting primary prevention to all women before and during pregnancy to avoid risk [7].

As the limitations of obstetric strategies to prevent threatened preterm birth become evident [7,8], primary prevention such as lifestyle change interventions have become increasingly compelling [9]. Recent evidence revealed that a healthy prenatal lifestyle (such as a healthy weight and a high-quality diet) was associated with a lower risk of preterm birth [10]. Pre-pregnancy and pregnancy lifestyle interventions targeting behaviors such as smoking, nutrition and physical activities are recommended by global [11] and national [12] bodies in response to the continuously observed relationships between lifestyle factors and preterm birth [13,14].

However, interventions targeting certain lifestyle factors associated with preterm birth have mixed results [8,15,16]. It is well-accepted that preterm birth is a syndrome resulting from multiple etiologic pathways, with many factors, including infection, inflammation, uteroplacental abnormity, stress, nutrition status and other immunologically mediating processes. It is difficult to distinguish the interactive impact of confounding factors. Furthermore, differences in intervention timing across studies also contribute to inconsistent conclusions about lifestyle interventions. Etiologic studies suggest the mechanisms leading to preterm birth are initiated in early pregnancy and progress with time [2]. Even similar exposures to risk factors (such as air pollution) at different times may result in different outcomes [17,18]. Failure to acknowledge the heterogeneity in the timing of interventions could contribute to misleading conclusions. Unfortunately, little attention has been paid to the role of timing lifestyle change interventions in preventing preterm birth.

Given the limited availability of evidence of lifestyle interventions for preventing preterm birth, the aim of this study was to explore associations between attendance, including the timing of attendance during pregnancy, at a brief antenatal lifestyle education seminar and preterm birth. We also examined the influence of demographic factors, health history (e.g., parity, CS history and pre-existing diabetes) and gestational conditions (e.g., GDM, pre-eclampsia/eclampsia, placenta implantation and cervical incompetence) on preterm birth to inform the development of tailored preterm birth prevention strategies.

## 2. Materials and Methods

**Figure 1:**
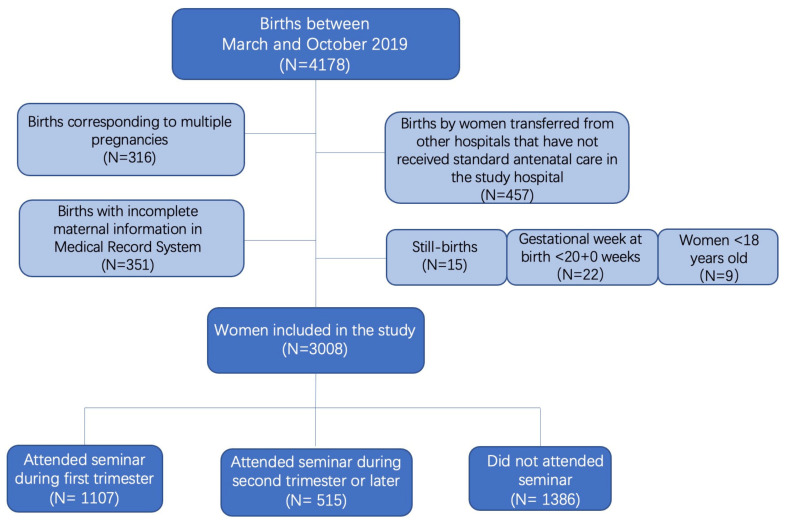
Study cohort flowchart.

This study was a retrospective analysis of the livebirth cohort of 2019 in one tertiary comprehensive hospital in Beijing, China. Records of births between March and Oct 2019 (*n* = 4178) in the study setting were reviewed. The study population was restricted to livebirths from singleton pregnancies. Women (*n* = 351) with key information (e.g., age, parity, gestational age at birth and neonatal birth condition) missing in the Electronic Medical Record System were excluded. Women who were transferred from another hospital (*n* = 457) were not included to avert the potential impact of different antenatal care practices. Women’s ages were restricted to ≥18 years, and the recorded gestational age at birth was ≥20 + 0 weeks. Figure 1 shows the patient selection process.

The antenatal lifestyle education intervention is a free, face-to-face, midwife-led group program about healthy lifestyle choices during pregnancy. The seminar commenced in 2018 to improve antenatal care in relation to birth outcomes. The two-hour seminar consisted of 60 min on gestational nutrition, 20 min on pregnancy weight management, 20 min on exercise during pregnancy and 20 min for questions and answers. Each component was based on the best available evidence, including the “2016 Pregnancy Women Nutrition Guidelines by the Chinese Nutrition Society [19]; the “Weight Gain During Pregnancy: Re-Examining the Guidelines” [20]; and the “ACOG Committee Opinion No. 650: Physical Activity & Exercise During Pregnancy & The Postpartum Period” [21]. Each Saturday, three trained midwives with more than ten years of clinical experience facilitated the seminar at a meeting hall in a hospital outpatient building. Slides and food models were used during the seminar, and relevant brochures were offered to participants.

During the first antenatal visit, women and their partners were encouraged by their doctors/midwives to attend the antenatal lifestyle seminar. As part of this brief introduction, women were told that the seminar was a free, optional resource and independent of their regular care. Women were offered a timetable; they were told that no appointment was necessary; and they only needed to swipe their medical identification card when entering the seminar room. The swipe system recorded the date of attendance into their Electronic Medical Record. Even though the seminar occurred every week, women were informed that they could only join once, mainly to ensure that more women could participate. In addition, written consent was obtained to allow the researchers to use their unidentifiable data for research purposes.

The primary outcome was preterm birth, defined as birth at <37 gestational weeks, in line with current WHO guidelines [22]. Gestational age was estimated by ultrasound or the last menstrual period if ultrasound dating was not available. Information on socio-demographics, maternal characteristics, gestational conditions and seminar attendance was extracted from the Electronic Medical Record System. Specifically, the socio-demographic and pre-existing maternal characteristics were women’s age at the first antenatal visit (<35 years versus ≥35 years); obesity at the first antenatal visit was defined as BMI ≥ 28 kg/m^2^ according to recommendations from the WHO Working Group on Obesity in China; parity; prior caesarean section (CS) or myomectomy (yes/no); prior cervical surgery referring to the loop electrosurgical excision procedure (LEEP); cone biopsy; laser surgery (yes/no); prior preterm birth (yes/no); pre-existing diabetes diagnosed using ICD-10 Code O24 (yes/no); chronic hypertension as pre-existing hypertension or developed before 20 weeks of pregnancy (yes/no); Polycystic Ovary Syndrome (PCOS) defined according to ICD-10 diagnosis Code E28.2 (yes/no); and the usage of assisted reproductive technology including in vitro fertilization or intracytoplasmic sperm injection (IVF/ICSI) (yes/no). During pregnancy, the covariates included gestational diabetes mellitus (GDM), gestational hypertension without significant proteinuria, pre-eclampsia or eclampsia, placenta implantation and the incompetence of cervix uteri, defined using ICD-10 Code O24, O13, O14, O15, O43 and N88.3. Whether women attended the seminar and gestational weeks at attendance (0 if absent, 1 if gestational age < 14 + 0 weeks (first trimester), and 2 if gestational age ≥ 14 + 0 (second trimester or later)) was also retrieved.

### 2.1. Statistical Analyses

Associations of all potential covariates with seminar attendance and preterm birth were examined with Fisher’s exact test. Significant associations with either attendance or preterm birth were selected for further stepwise binary logistic regression analyses to estimate odds ratios (ORs) and 95% CIs, taking into account different confounders (associated with preterm birth and antenatal education) and covariates (associated with preterm birth, not antenatal education). Firstly, the regression analysis was performed with preterm birth as the dependent variable and seminar attendance as the independent variable (Model A). Then, the analysis was adjusted for possible pre-existing confirmed confounders or covariates (Model B). Model C contained confirmed gestational factors, in addition to confounders or covariates included in Model B. Finally, subgroup analyses were performed among women with different maternal characteristics (e.g., age group, parity and pre-existing health conditions).

All information was drawn from the Electronic Medical Record System of the participating hospital using Excel and was then transferred to SPSS. All statistical analyses were performed using SPSS software (version 23.0, IBM, Armonk, NY, USA). The level of statistical significance was a *p*-value of <0.05, and the p-confidence interval was 95%. G*Power indicated that a minimum sample size of 2448 was required to detect a 0.1 or bigger effect size.

### 2.2. Ethical Approval

The study was approved by the Institutional Ethics Review Board (Approval No. M2018032).

## 3. Results

### 3.1. Maternal Characteristics and Antenatal Lifestyle Education Attendance

Of the total cohort of 3008 women, 1878 (62.4%) were less than 35 years of age, and 1073 (35.7%) were multiparas. Table 1 shows a detailed description of maternal characteristics for the cohort. For seminar attendance, 1107 (36.8%) women attended during the first trimester (9.9 ± 1.7 gestational weeks), 515 (17.1%) attended during the second trimester or later (19.0 ± 4.4 gestational weeks) and 1386 (46.1%) did not attend.

Table 1 also presents associations between attendance and women’s characteristics. Factors related to attendance were age (*p* < 0.05), parity (*p* < 0.001), prior CS or myomectomy (*p* < 0.001), assisted reproductive technology (*p* < 0.05), chronic hypertension (*p* < 0.05) and placenta implantation (*p* < 0.01). Including significant factors in the multivariate logistic regression model revealed that parity was the only independent influencing factor of women’s attendance. Compared to nulliparous women, the odds ratio for multiparas to attend the seminar in the first trimester was OR 0.37 (95% 0.30–0.46, *p* < 0.001), and attendance during the second trimester or later was 0.63 (95% 0.48–0.82, *p* < 0.01), indicating that multiparous women were more likely to attend at a later stage or to not attend.

### 3.2. Maternal Characteristics and Preterm Birth

From the cohort of 3008 births, the overall prevalence of preterm birth was 8.7%. There were significant differences in preterm birth rates between women who attended the seminar during the first trimester (5.8%), compared with 7.2% of women who attended during second trimester or later, and it was 11.5% among women who did not attend. Table 2 displays preterm birth rates stratified by women’s pre- and during-pregnancy characteristics. Women who had a preterm birth were more likely to be more than 35 years old (*p* < 0.01), to be obese (BMI > 25 kg/m^2^) (*p* < 0.001), to be multiparous (*p* < 0.01), to have had a prior CS or myomectomy (*p* < 0.001), to have had prior preterm birth or to have had pre-existing medical complications including hypertension (*p* < 0.001) and diabetes (*p* < 0.05). Women experiencing a preterm birth were more likely to have GDM (*p* < 0.05), preeclampsia or eclampsia, placenta implantation (*p* <0.001) or cervical incompetence (*p* < 0.001) (Table 2).

### 3.3. Association between the Timing of Seminar Attendance and Preterm Birth

As shown in Table 3, the unadjusted odds ratios for preterm birth among women who attended the seminar during the first trimester or later decreased by 53% (OR 0.47, 95% CI 0.35–0.64) compared with 41% for women who did not attend (OR 0.59, 95% CI 0.41–0.86) (Model A). Estimates changed slightly after adjusting confirmed pre-existing confounders and covariates including age, parity, prior CS/myomectomy, obesity, pre-existing diabetes, chronic hypertension and the usage of assisted reproductive technology (Model B): [aOR 0.54, 95% CI 0.39–0.73] for women attending during the first trimester and [aOR 0.58, 95% CI 0.40–0.86] for women attending ≥ 14 + 0 gestational weeks. When taking both confirmed pre-existing and gestational confounders and covariates (i.e., GDM, pre-eclampsia/eclampsia, placenta implantation and cervical incompetence) (Model C) into consideration, the estimates remained but at a lower level for women attending during the first trimester [aOR 0.61, 95% CI 0.43–0.85] and for women attending later [aOR 0.60, 95% CI 0.40–0.91].

### 3.4. Subgroup Logistic Regression

#### 3.4.1. Age

As presented in Table 3, the odds for preterm birth among women aged < 35 years were lower for those attending the seminar during the first trimester (aOR 0.45, 95% CI 0.30–0.66) and after 13 + 6 weeks (aOR 0.36, 95% CI 0.20–0.65). Similar outcomes were seen after adjusting for all confirmed confounders and covariates (Model B, C). For women aged 35 years or older, associations between first trimester attendance and preterm birth were only statistically significant in the unadjusted Model A and adjusted Model B. The association between preterm birth and second trimester or later attendance was not statistically significant with or without adjusting the confirmed confounders and covariates (Models A–C).

#### 3.4.2. Parity

As demonstrated in Table 3, attending the seminar either during the first trimester or later decreased preterm birth risk among nullipara women. However, women attending during their second trimester or later had the lowest risk based on the odds ratios in Models A–C. By contrast, for multiparous women, only first trimester attendance showed a statistically significant association with preterm birth in unadjusted Model A and in adjusted Model B (Table 3).

#### 3.4.3. Prior CS or Myomectomy

As presented in Table 3, for women who had a prior CS or myomectomy, first trimester attendance was significant in preventing preterm birth in unadjusted Model A and adjusted Model B. Attendance after the first trimester was only shown in adjusted Model B at a marginally statistically level (*p* = 0.04).

#### 3.4.4. Assisted Reproductive Technology

As shown in Table 3, the overall prevalence of preterm birth among women who received assisted reproductive technologies was 9.7%. Unexpectedly, women who did not attend the seminar had the lowest rate (8.4%) of preterm birth compared to women who attended during the first trimester (9.1%) or later (14.5%), although the difference was not significantly different (as shown in Table 3).

## 4. Discussion

To our knowledge, this study is one of the first to investigate the association between a brief antenatal lifestyle education intervention and preterm birth, and the very first to explore the effect of education timing among women with different maternal characteristics. We found that attending the lifestyle education seminar during pregnancy was associated with a decreased risk of preterm birth, and attending during first trimester had a better impact than later attendance.

Several reviews on the effects of antenatal lifestyle interventions to reduce preterm birth reported mixed results [8,23,24]. The inconsistent conclusions across studies may be due to the difficulty of controlling potential covariates [25], the collective impact of a set of behavioral risk factors on preterm birth [13,26] or the uncertainty regarding whether observed associations are causal. Preterm birth, as a public health issue, occurs in a larger social–cultural context [11]. The success of any preterm birth intervention strategy may therefore depend on its operating environment, including cultural, societal and economic aspects [12]. Nonetheless, the possibility of substantial reductions in preterm birth risk shown in our study merits further investment in lifestyle interventions.

The findings from this study highlight the critical role of the timing of lifestyle interventions. The latest evidence from environmental science and toxicology demonstrated that the impact of relevant exposures on preterm birth (e.g., air pollution [18], extreme heat weather [27] or drugs such as phenols [28]), varies according to when the exposure occurs. Weekly ‘critical window’ variables for certain exposures have been identified to guide preterm birth prevention practice [17,18]. Consistent with the notion of a critical window, we found that the association between attending the lifestyle seminar and preterm birth was modified by women’s attendance timing. However, unlike pollutants or drugs, women need time to respond to a lifestyle education program, which generates a delayed effect [7]. The uncertainty in lag-time may bias the effect estimates if using an overly discrete grouping as the variable [29]. Therefore, instead of grouping the exposure time weekly, we classified the exposure time into three groups: not exposed, exposed during the first trimester and exposed during the second trimester or later. This crude classification aligns with clinical antenatal practices, enhancing the likelihood of our findings being relevant for clinical staff and policy decision makers.

Our study demonstrates that different maternal characteristics are associated with attendance. Among younger or nulliparous women, any seminar attendance decreased the risk of preterm birth, but attendance later in pregnancy had a slightly better impact. Conversely, for older or multiparous women, early seminar attendance had a better impact, which was in adhering to current prevailing guidelines recommending early prematurity prevention for all pregnant women [6,11]. Such strategies could include the provision of the continuity of midwife care throughout pregnancy, labor and the postpartum period, which has been shown to reduce preterm birth by up to 24% [30]. The observed irregular phenomenon among young, nulliparous women illustrates the necessity of continuing to offer education at later trimesters, positing a particular noteworthy decay effect of antenatal lifestyle education among the younger, nulliparous population. The study also found that multiparous women tend not to attend antenatal education. There is consistent evidence that the current one-size-fits-all antenatal care model is not attractive for multiparous women [31,32]. Flexible and tailored antenatal care options need to be explored.

## 5. Strengths and Limitations

Our study has some strengths. First, the study included a relatively large sample of women with a wide range of maternal characteristics, which enabled us to conduct sub-group analyses. Second, we used real-world data drawn from the Electronic Medical Record System of a metropolitan hospital in China, thereby enhancing reliability and negating the effects of participant recall bias. Third, we used the stepwise binary logistic regression model, by which we classified the selected potential confounders and covariates according to time (pre-pregnancy or during pregnancy) and then added them gradually into the logistic regression models. The results generated from the chronological sequence models can enable clinicians to give more accurate information to women with various pre-pregnancy or gestational characteristics about the benefits of attending antenatal lifestyle education on preventing preterm birth and how the timing of attendance matters.

Limitations, however, need to be considered. Due to the retrospective cohort study design and the uncertainty regarding whether the observed associations are causal [2], the strength of recommending interventions is moderate [26]. Evidence has increasingly shown that factors contributing to preterm birth may be present prior to pregnancy [2]. This study, however, only explores the association between attending a brief lifestyle education seminar during pregnancy. We encourage future research to examine the efficacy of lifestyle education in preventing preterm birth using a broad timeframe [18]. The participating women were 18 years and older, all from one hospital, and the vast majority were Chinese, thereby limiting generalizability. Younger women are known to be at a greater risk of preterm birth than older women. It is necessary to confirm our findings in multi-center and multi-national studies with diverse samples.

## 6. Conclusions

Attending a brief antenatal lifestyle education seminar was associated with lower preterm birth risk. In the total cohort, first trimester attendance had an optimal impact on reducing preterm birth. For certain women (those who were older, who had a prior CS/myomectomy or who received IVF/ICSI), the current seminar made a very limited contribution to preventing preterm birth. More explorative research needs to be conducted to find out why that was the case.

The study findings can be used to counsel pregnant women or women planning for parenthood, as well as their families, about the benefits of attending education on preventing preterm birth and when to attend. Multiparous women tended not to attend the brief education seminar, so more flexible antenatal care options need to be explored to improve future clinical practice.

## Figures and Tables

**Table 1:** Maternal characteristics and seminar attendance.

Variables	Seminar Attendance			F/χ^2^	*p*
	Absence (*n* = 1386)	1st Trimester (*n* = 1107)	2nd Trimester or Later (*n* = 515)		
**Pre-existing factors (at first antenatal visit)**					
Age				7.580	0.023
<35	836 (60.3)	726 (65.6)	316 (61.4)		
≥35	550 (39.7)	381 (34.4)	199 (38.6)		
Obese				0.181	0.913
Yes	32 (2.3)	23 (2.1)	12 (2.3)		
No	1354 (97.7)	1084 (97.9)	503 (97.7)		
Parity				137.206	0.000
Nulliparous	752 (54.3)	851 (76.9)	331 (64.5)		
Multiparous	634 (45.7)	256 (23.1)	183 (35.5)		
Prior CS/myomectomy				59.889	0.000
Yes	321 (23.3)	124 (11.2)	94 (18.3)		
No	1065 (76.8)	983 (88.8)	421 (81.7)		
Prior cervical surgery				0.772	0.680
Yes	14 (1.0)	10 (0.9)	3 (0.6)		
No	1372 (99.0)	1097 (99.1)	512 (99.4)		
Prior preterm birth				3.706	0.157
Yes	19 (1.4)	7 (0.6)	4 (0.8)		
No	1367 (98.6)	1100 (99.4)	511 (99.2)		
Pre-existing diabetes				2.846	0.241
Yes	36 (2.6)	23 (2.1)	18 (3.5)		
No	1350 (97.4)	1084 (97.9)	497 (96.5)		
Chronic hypertension ^1^				6.754	0.034
Yes	52 (3.8)	26 (2.3)	24 (4.7)		
No	1334 (96.2)	1081 (97.7)	491 (95.3)		
PCOS ^2^				2.282	0.319
Yes	43 (3.1)	24 (2.2)	16 (3.1)		
No	1343 (96.9)	1083 (97.8)	499 (96.9)		
IVF/ICSI ^3^				7.570	0.023 *
Yes	143 (10.3)	154 (13.9)	62 (11.9)		
No	1243 (89.7)	953 (86.1)	453 (88.1)		
**Gestational factors**					
GDM ^4^				1.983	0.371
Yes	367 (26.5)	301 (27.2)	153 (29.7)		
No	1019 (73.5)	806 (72.8)	362 (70.3)		
Gestational hypertension without significant proteinuria				2.732	0.255
Yes	72 (5.2)	43 (3.9)	21 (4.1)		
No	1314 (94.8)	1064 (96.1)	494 (95.9)		
Preeclampsia or eclampsia				5.494	0.064
Yes	66 (4.8)	33 (3.0)	24 (4.7)		
No	1320 (95.2)	1074 (97.0)	491 (95.3)		
Placenta implantation				53.388	0.000
Yes	72 (5.2)	6 (0.5)	7 (1.4)		
No	1314 (94.8)	1101 (99.5)	508 (98.6)		
Incompetence of cervix uteri				0.441	0.802
Yes	26 (1.9)	21 (1.9)	12 (2.3)		
No	1360 (98.1)	1086 (98.1)	503 (97.7)		

^1^ Defined as pre-existing hypertension or developed before 20 weeks of pregnancy; ^2^ PCOS: Polycystic Ovary Syndrome; ^3^ IVF/ICSI: in vitro fertilization or intracytoplasmic sperm injection; ^4^ GDM: gestational diabetes mellitus. * *p* < 0.05

**Table 2:** Preterm birth rates stratified by maternal characteristics.

Variables	*n*	Preterm Birth *n* (%)	F/χ^2^	*p*
**Seminar attendance**				
Absence	1386	160 (11.5)	27.539	0.000
1st trimester	1107	64 (5.8)		
2nd trimester or later	515	37 (7.2)		
**Pre-existing factors (at first antenatal visit)**				
Age			9.423	0.002
<35	1878	140 (7.5)		
≥35	1130	121 (10.7)		
Obese			12.911	0.000
Yes	67	14 (20.9)		
No	2941	247 (8.4)		
Parity			16.342	0.000
Nulliparous	1935	138 (7.1)		
Multiparous	1073	123 (11.5)		
Prior CS/myomectomy			53.313	0.000
Yes	539	90 (16.7)		
No	2469	171 (6.9)		
Prior cervical surgery			1.295	0.202
Yes	27	4 (14.8)		
No	2981	257 (8.6)		
Prior preterm birth			4.903	0.027
Yes	30	6 (20.0)		
No	2978	255 (8.6)		
Pre-existing diabetes			4.758	0.029
Yes	77	12 (15.6)		
No	2931	249 (8.5)		
Chronic hypertension			62.830	0.000
Yes	102	31 (30.4)		
No	2906	230 (7.9)		
PCOS ^1^			2.256	0.133
Yes	83	250 (8.5)		
No	2925	11 (13.3)		
IVF/ICSI ^2^			0.592	0.442
Yes	359	35 (9.7)		
No	2649	226 (8.5)		
**Gestational factors**				
GDM ^3^			5.253	0.022
Yes	821	87 (10.6)		
No	2187	174 (8.0)		
Gestational hypertension without significant proteinuria			0.470	0.493
Yes	136	14 (10.3)		
No	2872	247 (8.6)		
Preeclampsia or eclampsia				
Yes	123	49 (39.8)	157.146	0.000
No	2885	212 (7.3)		
Placenta implantation			239.893	0.000
Yes	85	47 (55.5)		
No	2923	214 (7.3)		
Incompetence of cervix uteri			17.207	0.000
Yes	59	14 (23.7)		
No	2949	247 (8.4)		

^1^ PCOS: Polycystic Ovary Syndrome; ^2^ IVF/ICSI: in vitro fertilization or intracytoplasmic sperm injection; ^3^ GDM: gestational diabetes mellitus.

**Table 3:** Stepwise binary logistic regression of correlations between seminar attendance and preterm birth.

	Seminar Attendance	*n*	Preterm Birth Rate (%)	Model A Unadjusted ^1^	Model B Adjusted ^2^	Model C Adjusted ^3^
All cohorts		3008	8.7	*p* = 0.000	*p* = 0.000	*p* = 0.004
	Absence	1386	11.5	1	1	1
	1st trimester	1107	5.8	0.47 (0.35–0.64)	0.54 (0.39–0.73)	0.61 (0.43–0.85)
	2nd trimester or later	515	7.2	0.59 (0.41–0.86)	0.58 (0.40–0.86)	0.60 (0.40–0.91)
Age < 35		1878	7.5	*p* = 0.000	*p* = 0.000	*p* = 0.004
	Absence	836	10.8	1	1	1
	1st trimester	726	5.1	0.45 (0.30–0.66)	0.47 (0.31–0.71)	0.54 (0.35–0.86)
	2nd trimester or later	316	4.1	0.36 (0.20–0.65)	0.33 (0.18–0.62)	0.41 (0.21–0.79)
Age ≥ 35		1130	10.7	*p* = 0.021	*p* = 0.110	*p* = 0.331
	Absence	550	12.7	1	1	1
	1st trimester	381	7.1	0.52 (0.33–0.83)	0.60 (0.37–0.94)	0.68 (0.41–1.13)
	2nd trimester or later	199	12.1	0.94 (0.57–1.54)	0.94 (0.56–1.56)	0.91 (0.52–1.59)
Parity = 0		1935	7.1	*p* = 0.002	*p* = 0.004	*p* = 0.007
	Absence	752	9.7	1	1	1
	1st trimester	851	5.5	0.54 (0.37–0.80)	0.56 (0.38–0.82)	0.57 (0.38–0.86)
	2nd trimester or later	332	5.4	0.53 (0.31–0.91)	0.53 (0.31–0.91)	0.50 (0.28–0.88)
Parity ≥ 1		1073	11.5	*p* = 0.012	*p* = 0.014	*p* = 0.499
	Absence	634	13.7	1	1	1
	1st trimester	256	6.6	0.45 (0.26–0.77)	0.46 (0.26–0.80)	0.72 (0.35–1.19)
	2nd trimester or later	183	10.4	0.73 (0.43–1.23)	0.65 (0.37–1.14)	0.81 (0.40–1.39)
Prior CS/myomectomy		539	16.7	*p* = 0.016	*p* = 0.017	*p* = 0.433
	Absence	321	20.6	1	1	1
	1st trimester	124	10.5	0.45 (0.24–0.85)	0.47 (0.25–0.89)	0.86 (0.41–1.81)
	2nd trimester or later	94	11.7	0.51 (0.26–1.02)	0.48 (0.24–0.97)	0.59 (0.26–1.32)
IVF/ICSI ^4^		359	9.7	*p* = 0.380	*p* = 0.206	*p* = 0.173
	Absence	143	8.4	1	1	1
	1st trimester	154	9.1	1.09 (0.49–2.45)	1.20 (0.50–2.84)	1.47 (0.57–3.80)
	2nd trimester or later	62	14.5	1.85 (0.74–4.66)	2.32 (0.88–6.10)	2.79 (0.94–8.25)

^1^ Model A: unadjusted analysis.; ^2^ Model B: analysis adjusted for pre-pregnancy factors (i.e., age, obese, parity, prior CS or myomectomy, prior preterm birth, prior diabetes, chronic hypertension and the usage of assisted reproductive technology; ^3^ Model C: analysis adjusted for pre-existing maternal characteristics (same as Model B) and during-pregnancy factors (i.e., GDM, preeclampsia or eclampsia, placenta implantation or incompetence of cervix); ^4^ IVF/ICSI: in vitro fertilization or intracytoplasmic sperm injection.

## Data Availability

All data that support the findings of this study are available from the corresponding author (Jie Lu) upon reasonable request.
